# Changes in Gene Expression in the Larval Gut of *Ostrinia nubilalis* in Response to *Bacillus thuringiensis* Cry1Ab Protoxin Ingestion

**DOI:** 10.3390/toxins6041274

**Published:** 2014-04-03

**Authors:** Jianxiu Yao, Lawrent L. Buschman, Nanyan Lu, Chitvan Khajuria, Kun Yan Zhu

**Affiliations:** 1 Department of Entomology, 123 Waters Hall, Kansas State University, Manhattan, KS 66506, USA; E-Mails: jianxiu.yao@ag.tamu.edu (J.Y.); lbuschma@ksu.edu (L.L.B.); chitvank@gmail.com (C.K.); 2 Bioinformatics Center, Kansas State University, Manhattan, KS 66506, USA; E-Mail: nanyan@ksu.edu

**Keywords:** *Bacillus thuringiensis*, European corn borer, *Ostrinia nubilalis*, Cry1Ab protoxin, microarray, transcriptional response

## Abstract

We developed a microarray based on 2895 unique transcripts assembled from 15,000 cDNA sequences from the European corn borer (*Ostrinia* *nubilalis*) larval gut. This microarray was used to monitor gene expression in early third-instar larvae of *Bacillus thuringiensis* (Bt)-susceptible *O. nubilalis* after 6 h feeding on diet, with or without the Bt Cry1Ab protoxin. We identified 174 transcripts, for which the expression was changed more than two-fold in the gut of the larvae fed Cry1Ab protoxin (*p* < 0.05), representing 80 down-regulated and 94 up-regulated transcripts. Among 174 differentially expressed transcripts, 13 transcripts putatively encode proteins that are potentially involved in Bt toxicity, and these transcripts include eight serine proteases, three aminopeptidases, one alkaline phosphatase, and one cadherin. The expressions of trypsin-like protease and three aminopeptidase transcripts were variable, but two potential Bt-binding proteins, alkaline phosphatase and cadherin were consistently up-regulated in larvae fed Cry1Ab protoxin. The significantly up and down-regulated transcripts may be involved in Cry1Ab toxicity by activation, degradation, toxin binding, and other related cellular responses. This study is a preliminary survey of Cry1Ab protoxin-induced transcriptional responses in *O. nubilalis* gut and our results are expected to help with further studies on Bt toxin-insect interactions at the molecular level.

## 1. Introduction

The European corn borer, *Ostrinia nubilalis*, which primarily infests corn, is responsible for significant yield losses in North America [1]. In the United States alone, annual economic losses due to the direct damage and the costs of controlling this pest have been estimated to exceed $1 billion [2]. The insect-infested corn ears also experience substantial increases in mycotoxin contamination [3]. Transgenic corn hybrids, expressing Cry toxins encoded by genes derived from *Bacillus thuringiensis* (Bt), are one of the most successful technologies for controlling *O. nubilalis* under field conditions [4,5]. 

The mode of action of Bt toxins generally involves multiple steps, including: (1) solubilization of Bt crystals in insect midgut under certain pH conditions after ingestion; (2) activation of Bt protoxin to toxin by certain proteases (e.g., trypsins); (3) binding of activated toxin to a cadherin and/or a GPI-anchored protein (e.g., aminopeptidases, alkaline phosphatases); (4) insertion of the bound toxin oligomer into the lipid raft of the gut membrane to form pores; and (5) ultimately causing the gut cell to burst [6]. The binding of Bt toxin to cadherin has also been proposed to directly trigger intracellular signaling pathways involving stimulation of G proteins and adenylyl cyclase (AC), increasing cAMP levels, and activation of protein kinase A (PKA). The induction of AC and PKA results in cytological changes and cell blebbing, swelling, and lysis [7]. However, the signaling pathway model has been recently challenged by other scientists due to its poor support by experimental evidence [8]. Although the exact mode of action of Bt toxins has not been completely understood, it is clear that a number of genes expressed in insect gut are involved in Bt toxicity [6,8]. 

Cry toxins have been effective control agents with insecticidal specificity toward several insect pests in field, like *O. nubilalis* larvae. Therefore, these insects have the potential to develop resistance within a few generations if they are continuously exposed to Cry1Ab protoxin [9]. Even though there is no strong evidence related to field-evolved *O. nubilalis* resistance to Bt (Cry1Ab) corn, likely due to the implementation of high-dose/refuge resistance management strategies [10], reduced efficacy of Bt corn due to field-evolved resistance has been reported in some populations of other major corn pest species including *Busseola fusca* against Cry1Ab corn in South Africa, *P. gossypiella* against Cry1Ac cotton in India, *Diabrotica virgifera virgifera* against Cry3Bb corn, and *Spodoptera frugiperda* against Cry1F corn both in the United States [11]. Gassmann *et al.* [12] proposed that the insufficient planting of refuges and non-recessive inheritance of resistance may have contributed to resistance development in the field.

The mechanism of resistance to Bt toxin in insects is multifaceted, mirroring the complicated pore-formation mode of action involving multiple steps and gene products [8,13]. Our previous studies noted that transcript levels of several trypsin and trypsin-like protease genes were induced after the ingestion of Cry1Ab protoxin in larvae [14], and the activity of one soluble trypsin-like protease of a Dipel Bt-resistant strain of *O. nubilalis* was approximately half that of a susceptible strain [15]. The reduced trypsin-like activity was attributed to the reduced expression of *OnT23* in Bt-resistant *O. nubilalis* [16]. In *O. nubilalis*, Bt resistance has also been associated with decreased sensitivity or expression of Bt toxin-binding proteins, and increased expression of other intracellular defense proteins in the larval gut cells [7,9,17,18]. 

Microarray analysis is a widely used method to identify and analyze insect genes that are differentially expressed under specific conditions, such as insecticide exposures, microorganism infection, injury, *etc*. For example, microarrays have been used to identify gut gene expression responses of *Choristoneura* *fumiferana* under Bt Cry toxin exposure [19] and detoxification gene responses of *Anopheles gambiae* to insecticide exposure [20]. In order to provide a more comprehensive analysis, we developed a gut specific microarray for examining the transcriptional responses in Bt-susceptible *O. nubilalis* larvae after fed artificial diets containing Cry1Ab protoxin. Results from this research are expected to provide a platform for functional studies of toxin-insect interactions. 

## 2. Results and Discussion

### 2.1. Overview of Transcriptional Responses in O. nubilalis Larvae Fed Cry1Ab Protoxin

This study was to examine the transcriptional responses of the gut genes in *O. nubilalis* larvae fed Cry1Ab protoxin. We used the third-instar larvae that had been starved for 24 h to ensure they immediately started feeding on the experimental diets. Although we used the artificial diet containing the protoxin at the LC_50_ concentration (0.25 µg/mL diet) to feed the larvae, the protoxin did not cause any visible effect on the larvae because this LC_50_ value was determined based on a seven-day bioassay whereas the feeding duration in this study was only six hours. We used a feeding period of six hours since the larvae had stopped their feeding after they ingested the diet containing Cry1Ab protoxin. The use of the six-hour feeding period was to ensure sufficient amounts of Cry1Ab protoxin ingested by the larvae but minimum effect of starvation on gene expression when treated larvae stopped feeding after they ingested Cry1Ab protoxin. As shown by van Munster *et al.* [21], the majority of the genes had altered transcriptional levels at five hours when they analyzed the dissected midgut of *C. fumiferana* larvae 15 min, 2 h, 5 h, and 24 h post Cry1Ab protoxin ingestion at a single sublethal concentration.

**Figure 1:**
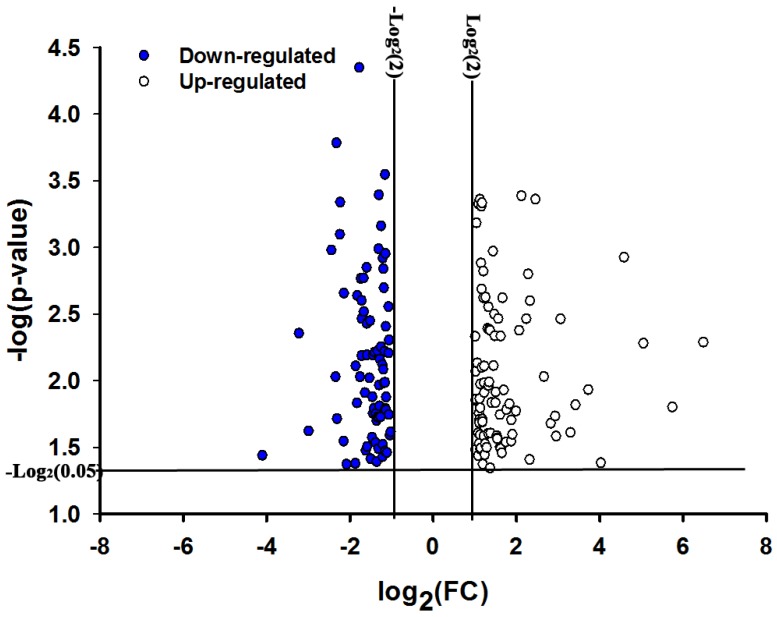
Transcript expression profiles from the gut of *O. nubilalis* larvae fed Cry1Ab protoxin. The transcriptional responses were compared between the larvae fed a diet containing Cry1Ab protoxin (treatment) and larvae fed a normal diet (control) by using one-way ANOVA (*p* < 0.05) and the Benjamini-Hochberg multiple testing correction (*q* < 0.05). Finally, 174 transcripts were identified as differentially expressed based on the cutoff of the fold change (FC) at ≥2.0. The *p*-value and fold change of each transcript was transformed to negative log and log_2_ scale, respectively, in the plot. The solid data points represent down-regulated gut transcripts (total 80) and clear data points represent up-regulated transcripts (total 94).

By using the Agilent custom microarray that contained 12,297 probes representing 2895 unique transcripts from the gut of *O. nubilalis* larvae, we identified 758 probes representing174 transcripts as differentially expressed in the larvae fed diet containing Cry1Ab protoxin (*p* < 0.05, expression ratio or fold change ≥2 fold [22]. These transcripts represent 80 down-regulated and 94 up-regulated genes (Figure 1). Among these 174 differentially expressed transcripts, 119 had BLAST results (*E*-value < 1.0e−3) (Table 1), whereas 56 did not after running global BLAST and CDS (conserved domain search) searches in the National Center for Biotechnology Information (NCBI) (Supplementary Table S1). This analysis was limited to those transcripts/proteins that are functionally-annotated in the database. Only 68% of the 174 gut transcripts that were differentially expressed in response to the ingestion of Cry1Ab1 protoxin had homolog descriptions in the database.

Among the 119 transcripts with BLAST hits, 14 were potentially involved in Bt toxin solubilization, activation, degradation; five were involved in Bt toxin binding; three were involved in signal transduction; seven served as transporters; five appeared to be transcription factors and might influence gene expression; 49 were involved in diverse metabolic processes including xenobiotic, amino acid, carbohydrate, lipid, and chitin metabolisms; three were related to anti-bacterial proteins; and the remaining 23 were involved in other diverse functions (Table 1). Among the 49 transcripts related to various metabolisms, approximately 81% were down-regulated, probably caused by the reduced food uptake due to the ingestion of Cry1Ab protoxin. Eleven transcripts potentially involved in carbohydrate metabolic pathways, including one α-amylase, two enolases, one glucose phosphate dehydrogenase, one glycoside hydrolase, one hydroxybutyrate dehydrogenase, and five glucosyltransferases, were down-regulated. Similarly, seven transcripts involved in lipid metabolic pathways, such as alkaline ceramidase-like enzyme, lipase, and desaturase, were also down-regulated.

**Table 1:** Summary of 119 significantly differentially expressed (fold change ≥2.0 and *p* < 0.05) transcripts with BLAST results from *O. nubilalis* larvae in response to the ingestion of Cry1Ab protoxin.

EST ID	NCBI EST database ID	Gene Homologs	Homolog GenBank Accession No.	Fold Change ± SE *
**Bt toxin solubilization, activation, degradation or sequestration**				
contig [0243]	GH998064.1	trypsin precursor	AFM77760.1	−2.69 ± 0.29
contig [0389]	GH998056.1	serine protease	AFM77769.1	8.39 ± 0.02
contig [0770]	GH997442.1	trypsin-like serine protease	AFM77762.1	3.16 ± 0.14
contig [1207]	GH997507.1	serine protease	AFM77770.1	7.67 ± 0.26
contig [3704]	GH999118.1	trypsin-like serine protease	AFM77754.1	6.36 ± 0.04
contig [4768]	GH998250.1	trypsin-like serine protease	AFM77753.1	10.75 ± 0.12
contig [5740]	GH999046.1	chymotrypsin-2 (chymotrypsin ii)	AFM77774.1	3.26 ± 0.19
ECB-C-18_B11	GH994018.1	silk gland derived serine protease	AAR98920.2	−2.63 ± 0.04
contig [0115]	GH997328.1	esterase FE4-like (*Bombyx mori*)	XP_004924612.1	3.69 ± 0.13
contig [3820]	GH999448.1	carboxylesterase (*Helicoverpa armigera*)	ADE05548.1	−2.21 ± 0.12
J-ECB-07_G03	GH991809.1	carboxylesterase (*Loxostege sticticalis*)	ACA50924.1	3.58 ± 0.06
J-ECB-09_D02	GH992373.1	carboxylesterase (*Spodoptera litura*)	AEJ38204.1	−3.31 ± 0.22
ECB-17_F12	GH998536.1	carboxylesterase (*Helicoverpa armigera*)	ADD97156.1	3.99 ± 0.06
ECB-27_F04	GH999378.1	carboxyl/cholinesterase 4A (*Bombyx mori*)	NP_001116814.1	−2.83 ± 0.11
**Potential Bt toxin binding partners**				
J-ECB-25_B09	GH990771.1	cadherin-like protein	ACK37450.1	2.85 ± 0.20
ECB-V-05_D12	GH994582.1	aminopeptidase n3	AEO12689.1	−2.55 ± 0.23
contig [4776]	GH998970.1	aminopeptidase n2	ACJ64828.1	2.17 ± 0.09
contig [4879]	GH997475.1	aminopeptidase n8	ACV04931.1	−2.31 ± 0.12
contig [5858]	GH998639.1	membrane-bound alkaline phosphatase (*Ostrinia furnacalis*)	AEM43806.1	2.23 ± 0.06
**Signal transduction**				
contig [0492]	GH998299.1	caspase-4 (*Lymantria* *monacha*)	AEK20829.1	2.36 ± 0.03
ECB-10_C01	GH997883.1	pyridoxal kinase (*Bombyx* *mori*)	NP_001037440.1	−2.99 ± 0.10
contig [5143]	GH995296.1	ctl2 antioxidant enzyme (*Aedes* *aegypti*)	XP_001661235.1	2.32 ± 0.04
**Transporter**				
contig [0814]	GH998546.1	sodium-bile acid cotransporter (*Danaus plexippus*)	EHJ73754.1	−5.03 ± 0.05
contig [1314]	GH993616.1	potassium coupled amino acid transporter (*Manduca* *sexta*)	AAF18560.1	−4.68 ± 0.16
contig [4763]	GH998142.1	sodium-bile acid cotransporter (*Aedes* *aegypti*)	XP_001662576.1	−4.39 ± 0.10
contig [5743]	GH993678.1	amino acid transporter (*Bombyx* *mori*)	NP_001124343.1	−5.40 ± 0.13
ECB-21_C09	GH998857.1	sugar transporter (*Danaus plexippus*)	EHJ73890.1	−2.46 ± 0.10
J-ECB-39_E12	GH992066.1	sugar transporter (*Culex* *quinquefasciatus*)	XP_001862938.1	−2.65 ± 0.25
J-ECB-55_E04	GH988996.1	monocarboxylate transporter (*Bombyx mori*)	XP_004927805.1	−3.40 ± 0.10
**Transcription factor and gene expression**				
contig [3833]	GH993952.1	DNA-binding nuclear protein p8 (*Simulium* *guianense*)	ACH56888.1	4.89 ± 0.09
contig [4800]	GH998367.1	endonuclease-reverse transcriptase (*Bombyx* *mori*)	ADI61826.1	2.49 ± 0.04
ECB-V-26_F03	GH996285.1	histone H3.2-like (*Meleagris* *gallopavo*)	XP_003202254.1	−2.04 ± 0.02
contig [3869]	GH997175.1	cellular repressor of E1A-stimulated genes 1 (*Tribolium* *castaneum*)	XP_972946.1	−3.16 ± 0.06
contig [5038]	GH991382.1	MluI cell cycle box (MCB) Binding Factor 2 (*Samia* *cynthia*)	BAA34219.1	2.41 ± 0.11
**Metabolism**				
**Xenobiotics metabolism**				
contig [0004]	GH992504.1	glutathione *S*-transferase (*Choristoneura* *fumiferana*)	AAF23078.1	−3.53 ± 0.45
contig [2246]	GH991501.1	glutathione *S*-transferase 16 (*Helicoverpa* *armigera*)	ACU09495.1	−2.59 ± 0.57
contig [0012]	GH987677.1	microsomal glutathione transferase (*Heliothis* *virescens*)	ADH16761.1	−2.19 ± 0.13
ECB-C-03_D08	GH992802.1	cytochrome P450 monooxygenase cyp6ab4 (*Bombyx* *mandarina*)	NP_001073135.1	−2.70 ± 0.08
contig [5080]	GH996933.1	cytochrome P450 monooxygenase cyp4m5(*Bombyx* *mori*)	NP_001103833.1	2.81 ± 0.11
J-ECB-21_A02	GH988690.1	aliphatic nitrilase (*Bombyx* *mori*)	NP_001165388.1	−2.43 ± 0.43
**Lipid metabolism**				
J-ECB-35_D11	GH990122.1	alkaline ceramidase-like isoform 1 (*Bombus* *terrestris*)	XP_003393007.1	−3.09 ± 0.37
contig [0029]	GH998728.1	acidic lipase (*Helicoverpa armigera*)	AFI64313.1	−2.08 ± 0.04
contig [0140]	GH998810.1	neutral lipase (*Helicoverpa armigera*)	AFI64310.1	−4.47 ± 0.11
contig [1081]	GH998825.1	neutral lipase (*Helicoverpa armigera*)	AFI64314.1	−2.58 ± 0.05
**Lipid metabolism**				
contig [1486]	GH997709.1	C-5 sterol desaturase erg32-like (*Bombyx mori*)	XP_004922936.1	−4.96 ± 0.13
contig [1897]	GH997709.1	C-5 sterol desaturase-like (*Acyrthosiphon* *pisum*)	XP_001947459.1	−4.48 ± 0.02
J-ECB-11_B07	GH988922.1	fatty acid-binding protein, muscle-like isoform 2 (*Nasonia vitripennis*)	XP_001608053.1	−2.47 ± 0.10
**Carbohydrate metabolism**				
contig [4242]	GH998158.1	alpha-amylase 2 (*Diatraea* *saccharalis*)	AAP97393.1	−2.21 ± 0.03
contig [4425]	GH988573.1	enolase (*Antheraea* *pernyi*)	ADO40102.1	−2.36 ± 0.25
ECB-V-12_H04	GH995176.1	enolase (*Spodoptera litura*)	AGQ53952.1	−2.26 ± 0.02
ECB-28_F02	GH999466.1	glucose phosphate dehydrogenase (*Axia* *margarita*)	ADW85328.1	−3.05 ± 0.27
contig [5232]	GH987506.1	glycoside hydrolases (*Aedes* *aegypti*)	XP_001659854.1	−2.31 ± 0.04
contig [4123]	GH990084.1	glucose and ribitol dehydrogenase-like (*Bombyx mori*)	XP_004922759.1	−2.28 ± 0.10
ECB-V-05_G12	GH994609.1	UDP-glycosyltransferase UGT33J1 (*Helicoverpa armigera*)	AEW43118.1	−2.27 ± 0.07
ECB-V-08_G03	GH994852.1	UDP-glycosyltransferase UGT33F1 (*Helicoverpa armigera*)	AEW43115.1	−2.25 ± 0.07
ECB-12_E11	GH998082.1	UDP-glycosyltransferase UGT40K1 (*Bombyx mori*)	AEW43171.1	−3.62 ± 0.27
ECB-V-19_F07	GH995711.1	glycosyltransferase 2 (*Chilo suppressalis*)	AGG36457.1	−2.42 ± 0.02
ECB-V-22_H08	GH995978.1	UDP-glycosyltransferase UGT40K1 (*Bombyx mori*)	AEW43171.1	−2.80 ± 0.07
**Amino acid metabolism**				
contig [4515]	GH987646.1	gamma-glutamyl hydrolase A-like (*Bombyx mori*)	XP_004931467.1	−2.50 ± 0.03
J-ECB-24_G10	GH990570.1	methyltransferase (*Mesobuthus* *caucasicus*)	CAE53466.1	2.14 ± 0.03
contig [5690]	GH988024.1	farnesoic acid O-methyltransferase (*Bombyx mori*)	AGS17914.1	2.92 ± 0.07
contig [1237]	GH989714.1	farnesoic acid O-methyltransferase (*Bombyx mori*)	AGS17915.1	2.90 ± 0.05
J-ECB-30_A09	GH987906.1	farnesoic acid O-methyltransferase (*Bombyx mori*)	AGS17914.1	2.91 ± 0.08
contig [5679]	GH988679.1	phosphoserine aminotransferase (*Antheraea* *pernyi*)	ADO79970.1	−2.18 ± 0.08
ECB-09_B04	GH997795.1	asparagine synthetase (*Bombyx* *mori*)	NP_001037414.1	−2.38 ± 0.29
**Gut chitin metabolism**				
contig [0188]	GH997506.1	chitinase (*Ostrinia nubilalis*)chtinase 8 (*Drosophila* *melanogaster*)	ADB85578.1	−2.74 ± 0.23
ECB-V-28_H03	GH996480.1	chitin synthase (*Ostrinia furnacalis*)	ABB97082.1	2.16 ± 0.02
ECB-C-05_D05	GH992955.1	glucosamine-fructose-6-phosphate aminotransferase 2 (*Culex quinquefasciatus*)	XP_001848160.1	−2.02 ± 0.01
**Other metabolic enzymes**				
contig [0077]	GH998660.1	caboxypeptidase 4 (*Mamestra configurata*)	ACN69214.1	−2.25 ± 0.04
contig [0009]	GH992549.1	carboxypeptidase (*Bombyx mori*)	AFD99126.1	−2.19 ± 0.05
contig [0019]	GH998697.1	plasma glutamate carboxypeptidase, partial (*Spodoptera exigua*)	AFM38216.1	−3.16 ± 0.19
J-ECB-33_G12	GH989302.1	juvenile hormone epoxide hydrolase-like protein 1 (*Bombyx mori*)	NP_001159617.1	−3.00 ± 0.06
contig [0557]	GH998460.1	juvenile hormone epoxide hydrolase (*Spodoptera exigua*)	ABD85119.1	−2.17 ± 0.02
contig [1953]	GH997798.1	NADP-dependent oxidoreductase (*Bombyx* *mori*)	NP_001091765.1	−3.27 ± 0.09
contig [3531]	GH995654.1	aldo-keto reductase (*Aedes* *aegypti*)	XP_001648461.1	−2.23 ± 0.03
contig [4521]	GH989023.1	aldo-keto reductase (*Bombyx* *mori*)	ADQ89807.1	−4.40 ± 0.60
J-ECB-37_E05	GH991174.1	oxidoreductase (*Acromyrmex* *echinatior*)	EGI66780.1	−2.37 ± 0.05
contig [4410]	GH994481.1	methionine-R-sulfoxide reductase B1-like isoform X2 (*Bombyx mori*)	XP_004924661.1	−2.46 ± 0.03
contig [3814]	GH994966.1	alcohol dehydrogenase (*Bombyx mori*)	ADM32152.1	−2.07 ± 0.03
gi_133906638	EL929475.1	retinol dehydrogenase 11-like (Bombyx mori)	XP_004926801.1	−3.61 ± 0.62
contig [5542]	GH997904.1	acetyltransferase 1 (*Danaus plexippus*)	EHJ65205.1	−2.98 ± 0.27
J-ECB-39_F07	GH992097.1	cytidylate kinase (*Bombyx* *mori*)	NP_001040356.1	−2.73 ± 0.06
BM2_M13R_B12	GH992538.1	estradiol 17-beta-dehydrogenase 8-like isoform X1 (*Bombyx mori*)	XP_004928638.1	−2.09 ± 0.07
**Anti-bacterial related protein**				
J-ECB-60_D07	GH987186.1	antibacterial protein (*Heliothis* *virescens*)	ACI02333.1	2.84 ± 0.07
gi_133905829	EL928679.1	hinnavin II antibacterial peptides (*Pieris* *rapae*)	AAT94287.1	7.13 ± 0.22
contig [2223]	GH996406.1	peptidoglycan recognition protein C (*Ostrinia nubilalis*)	ADU33186.1	5.04 ± 0.07
**Others**				
contig [0347]	GH987380.1	fatty acid binding protein 1 (*Manduca sexta*)	P31416.1	6.89 ± 0.35
ECB-V-18_A08	GH995588.1	Fatty acid-binding protein 2 (*Danaus plexippus*)	EHJ79280.1	−4.19 ± 0.32
contig [0028]	GH999333.1	cytochrome b5 (*Helicoverpa armigera*)	ADU02195.1	−2.70 ± 0.41
contig [2048]	GH994666.1	cytochrome b561 domain-containing protein 2-like (*Bombyx mori*)	XP_004933387.1	2.26 ± 0.03
contig [4527]	GH989618.1	cytochrome b561 domain-containing protein 1-like (Bombyx mori)	XP_004928254.1	−7.89 ± 0.34
ECB-11_E06	GH997996.1	peroxisomal membrane protein 11C-like (*Bombyx mori*)	XP_004925254.1	−3.51 ± 0.23
gi_133907290	EL930112.1	interferon-induced very large GTPase 1-like (*Danio rerio*)	XP_005163746.1	4.37 ± 0.27
contig [0566]	GH993617.1	fatty acid binding protein (*Spodoptera litura*)	AEH16743.1	−2.17 ± 0.03
contig [1640]	GH991677.1	fatty acid-binding protein, adipocyte-like (*Bombyx mori*)	XP_004930401.1	−2.99 ± 0.14
contig [2896]	GH987914.1	lipid storage droplet protein 2 (*Manduca sexta*)	AEJ33049.1	2.28 ± 0.10
contig [0407]	GH997662.1	sensory appendage protein 3 (*Manduca* *sexta*)	AAF16707.1	−17.04 ± 3.80
J-ECB-08_B02	GH991953.1	putative chemosensory protein (*Sesamia inferens*)	AGY49266.1	−9.26 ± 0.49
ECB-19_G03	GH998716.1	nose resistant to fluoxetine protein 6-like (*Bombyx mori*)	XP_004929562.1	2.75 ± 0.01
ECB-V-07_D03	GH994735.1	serine-rich adhesin for platelets-like (*Ceratitis capitata*)	XP_004536543.1	2.66 ± 0.05
contig [5724]	EL928855.1	silk protein P25 (*Corcyra cephalonica*)	ACX50393.1	33.34 ± 1.92
contig [4952]	GH988655.1	fibroin light chain (*Haritalodes derogata*)	AFS32690.1	53.91 ± 1.63
ECB-02_H03	GH997311.1	saposin-like protein (*Bombyx mori*)	ADU03994.1	−2.55 ± 0.35
contig [5293]	GH997917.1	trypsin inhibitor (*Bombyx* *mori*)	NP_001037044.1	90.23 ± 1.47
contig [5386]	GH996141.1	leukocyte surface antigen CD53-like isoform X5 (*Bombyx mori*)	XP_004926002.1	2.33 ± 0.06
ECB-C-04_H06	GH992916.1	tetraspanin D107 (*Plutella xylostella*)	BAD52262.1	2.25 ± 0.03
contig [5414]	GH997359.1	polyubiquitin-C-like isoform X1 (*Musca domestica*)	XP_005179902.1	2.74 ± 0.09
contig [1085]	GH992931.1	larvae cuticle protein (*Choristoneura fumiferana*)	AFC88812.1	−4.92 ± 0.36
J-ECB-12_F09	GH989631.1	globin 1 (*Bombyx mori*)	NP_001136083.1	−2.54 ± 0.40
J-ECB-29_G03	GH992468.1	IST1 homolog (*Bombyx mori*)	XP_004931988.1	2.18 ± 0.07
J-ECB-32_D06	GH988574.1	tetratricopeptide repeat protein 27-like (*Bombyx mori*)	XP_004930370.1	−2.44 ± 0.01
J-ECB-47_A02	GH990851.1	hepatocyte growth factor-regulated tyrosine kinase substrate-like (*Bombyx mori*)	XP_004932480.1	3.09 ± 0.62
**Others**				
gi_133905779	EL928629.1	pantetheinase (*Mamestra configurata*)	AEA76314.1	−3.36 ± 0.13
ECB-19_B09	GH998666.1	vanin-like protein 2-like (*Bombyx mori*)	XP_004928912.1	2.56 ± 0.03
J-ECB-14_H06	GH990255.1	circadian clock-controlled protein (*Harpegnathos saltator*)	EFN85083.1	2.27 ± 0.10
ECB-V-26_F03	GH996285.1	Histone H3c (*Culex quinquefasciatus*)	XP_001862696.1	−2.04 ± 0.02
J-ECB-07_A03	GH991471.1	circadian clock-controlled protein-like (*Bombyx mori*)	XP_004932669.1	5.02 ± 0.46
J-ECB-39_H09	GH992207.1	extracellular domains-containing protein CG31004-like isoform X2 (*Bombyx mori*)	XP_004925419.1	2.15 ± 0.02
ECB-V-25_C10	GH996179.1	conserved hypothetical protein (*Culex quinquefasciatus*)	XP_001845252.1	2.29 ± 0.08

* The fold change and its standard errors (SE) were calculated based on five probes of the same transcript in the *O. nubilalis* larvae fed the artificial diet containing Cry1Ab protoxin and in the control larvae fed artificial diet without the protoxin. The symbol “−” before the fold change indicates down regulation of the transcript in the gut of *O. nubilalis* larvae fed on Cry1Ab protoxin. The values of the fold change in the last column are bolded if they are >10-fold.

### 2.2. Transcriptional Responses of Genes Potentially Involved in Protoxin Activation or Degradation

**Figure 2:**
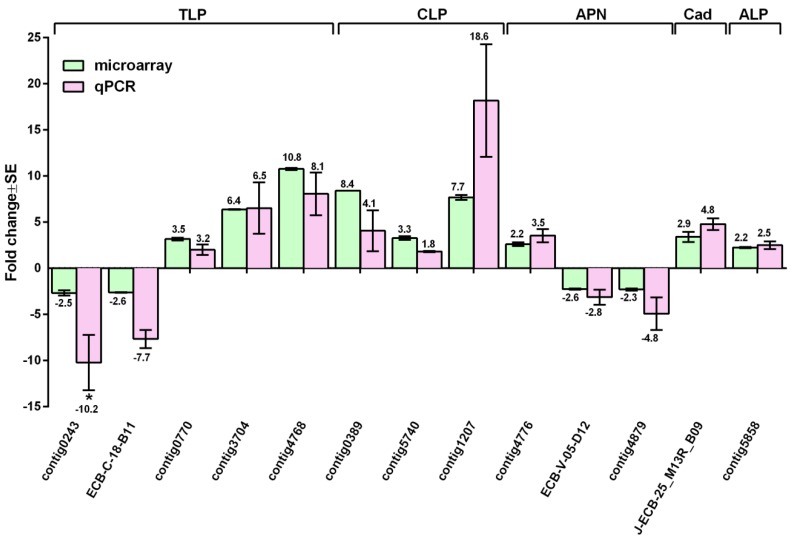
Validation of microarray data using RT-qPCR. Microarray (spotted bar) and RT-qPCR (outlined diamond bar) analyses of 13 differentially regulated transcripts, sequentially, including those encoding putative trypsin and trypsin-like serine protease transcripts (TLP) (EST ID: contig [4786], contig [3704], contig [0770], contig [0243], ECB-C18-B11), chymotrypsin and chymotrypsin-like serine protease transcripts (CLP) (EST ID: contig [0389], contig [1207], contig [5740]), aminopeptidase (APN) (EST ID: contig [4776], contig [4879], ECB-V05_D12), cadherin (Cad) (EST ID: J-ECB-25B09), and alkaline phosphatase (ALP) (EST ID: contig [5858]). The fold change of each transcript in the microarray (*p*-value < 0.05, and fold change cut off ≥2 folds) and RT-qPCR analyses (*p*-value ≤ 0.05) are marked on the top of each column. The symbol “*” indicates that RT-qPCR of contig [0243] did not show significant difference between the Cry1Ab protoxin and no protoxin treatments (*p* > 0.05).

When a Cry protoxin is ingested by insects, it is cleaved by proteases in the gut to yield an active toxin [23,24]. This produces an activated toxin monomer that can interact with the insect gut receptors. Thus, Bt protoxin activation or degradation are primary factors influencing protoxin Bt toxicity after ingestion [25,26,27]. In this study, we found that serine proteases constituted the most abundant group of transcripts (8) that were differentially expressed when *O. nubilalis* larvae were fed Cry1Ab protoxin (Table 1). These include five trypsin and trypsin-like transcripts (contig [4768], contig [0389], contig [1207], contig [0243, and ECB-C-18_B11) and three chymotrypsin and chymotrypsin-like transcripts (contig [3704], contig [5740], and contig [0770]), in which three trypsins and three chymotrypsins were up-regulated. One trypsin-like protease transcript (contig [4768]) showed >10-fold up-regulation based on the microarray data. This transcript may be directly involved in proteolytic activation of Cry1Ab protoxin to toxin after protoxin ingestion [26]. Interestingly, we also found that one trypsin inhibitor transcript (contig [5293]) was up-regulated by 90-fold based on the microarray data. This trypsin inhibitor gene may be involved in insect defense against Cry1Ab intoxication by inhibiting proteolytic activation of protoxin to toxin. The transcriptional changes of chymotrypsin (contig [3704], contig [5740], contig [0770]) and trypsin (contig [4768], contig [0389], contig [1207], contig [0243], and ECB-C-18_B11) transcripts were also validated by reverse transcription quantitative PCR (RT-qPCR), in which most transcript change ratios are consistent with microarray data, except for one trypsin transcript (contig [0243]) (Figure 2). These serine proteases could potentially be involved in the proteolysis of Cry1Ab protoxin either for activation or degradation of the protoxin. 

Li *et al.* [16] reported that the larvae of the KS-SC Dipel Bt-resistant strain of *O. nubilalis* had relatively lower trypsin activity than the susceptible strain. One trypsin transcript (OnTry23) was expressed at lower levels in the resistant strain relative to a susceptible strain, but another transcript (OnTry25) was not significantly different for the two strains. This implies that OnTry23 may be involved in resistance to Dipel Cry protoxins by decreasing protoxin activation in resistant strains of *O. nubilalis*. In a resistant strain of *Plodia interpunctella*, the lack of a major gut protease activity, PiT2 (accession No: AF064525), was responsible for about 90% of the resistance to Cry1Ab protoxin in a *B. thuringiensis* subsp. *entomocidus*-resistant colony [26,27]. Our previous studies showed that OnTry5 (contig [4786]), OnTry6 (contig [3704]), and OnTry14 (contig [0770]) shared 78, 69, and 68% amino acid sequence identities, respectively, with PiT2, and were clustered with PiT2 in phylogenetic analysis [14]. Thus, the relatively high similarity of these *O. nubilalis* trypsin transcripts with PiT2 suggests they may have a similar role in protoxin activation in *O. nubilalis*.

In lepidopteran larval gut, trypsins appear to function mainly in Bt protoxin activation, whereas chymotrypsins appear to be more important in toxin degradation [25]. In our study, we observed the up-regulation of three transcripts (contig [3704], contig [5740], contig [0770]) putatively encoding chymotrypsins. Although our results do not provide direct evidence for the involvement of these trypsin and chymotrypsin genes in the activation or degradation of Cry1Ab protoxin, the significant changes in expression of these genes in the larval gut in response to Cry1Ab protoxin ingestion suggests their involvement in Bt protoxin activation or degradation. Further studies are needed to confirm these results at the protein level by identifying them by MS/MS and quantifying their relative activities by blotting using chymotrypsin and trypsin-specific reagents, and clarify their roles probably by using RNA interference (RNAi).

### 2.3. Transcriptional Responses of Genes Potentially Involved in Toxin Binding

In the pore-formation model of Bt mode of action, the active monomeric toxin binds to cadherin, resulting in further toxin processing to form toxin oligomers, possibly involving *N*-acetylgalactosamine residues on *N*-aminopeptidases (APNs) and alkaline phosphatases (ALPs) [13]. In relation to this model, we found a 2.9-fold up-regulation of a cadherin-like transcript (J-ECB-25-B09) in Cry1Ab protoxin-fed *O. nubilalis* larvae. This cadherin-like protein showed 60% amino acid sequence identity with the cadherin of *Manduca sexta*, which has been known to be associated with Cry1Ab binding and cytotoxicity [28]. In fact, a cadherin-like protein has been shown to act as a receptor and is involved in Cry1Ab toxicity in *O. nubilalis* [29]. However, it is unclear why this cadherin-like transcript was up-regulated in response to CryAb1 ingestion in *O. nubilalis* larvae. 

In this study, we also found differential expressions of three APN and one ALP transcripts in the larvae fed Cry1Ab protoxin (Table 1). The ALP transcript (contig [5858]) and one of the three APN transcripts (contig [4776]) were up-regulated by ~2 fold, while the other two APN transcripts (contig [4879] and ECB-V-05-D12) were down-regulated by ~2 fold. These changes in the microarray data (J-ECB-25_B09, contig [5858], contig [4776], contig [4879] and ECB-V-05-D12) were further confirmed by RT-qPCR (Figure 2). Microarray and RT-qPCR had the same transcript change pattern, except that the change was greater in the RT-qPCR analysis than in the microarray analysis (Figure 2).

APNs have also been proposed as receptors for Bt Cry toxin in several lepidopteran species, such as *M. sexta* [30], Asian corn borer (*O. furnacalis*) [31], sugarcane borer (*Diatraea saccharalis*) [32], and cotton leafworm (*Spodoptera litura*) [33]. The injection of dsRNA for an APN gene in *S. litura* resulted in reduced transcript levels and decreased susceptibility to Cry1C toxin) [33]. Moreover, the APN-N1 gene was not expressed in a lab-selected Cry1C resistant colony of *S. exigua*) [34]. In our recent study using RNA interference suggested that an aminopeptidase-P like gene could be involved in binding Cry1Ab toxin in larval midgut of *O. nubilalis*) [35]. However, the up-regulated APN transcript in this study (contig [4776]) belongs to the APN2 group. Further research is needed to better understand its role in Cry1Ab toxicity in *O. nubilalis*. 

### 2.4. Transcriptional Responses of Genes Potentially Involved in Larval Defense

The ingestion of Cry toxin by insects can trigger transcription changes of the genes involved not only in Bt toxicity, but also in defense and repair mechanisms. For example, the ingestion of Cry toxins destroys the epithelial membrane of the insect gut, which leads to the leak of the gut contents into the hemolymph and promoting septicemia [36]. Therefore, under Cry toxin exposure, larvae may invoke the mechanisms that reduce the damage and support a functional gut system. 

In this study, we found a five-fold increased expression of a peptidoglycan recognition protein (PGRP) transcript (EST id: contig [2223]) in the gut of larvae fed Cry1Ab toxin (Table 1). We also found that two antimicrobial peptide transcripts, including an antibacterial protein (J-ECB-60_D07) and a hinnavin II antibacterial peptide (gi_133905829), that were up-regulated by ~3 and 7 fold, respectively. The up-regulation of these transcripts may imply the involvement of these genes in larval defense against the septicemia that results from pore formation during Cry toxin activity [36,37]. In addition, three transcripts, encoding proteins similar to caspase-4 and catalase-2 (ctl-2) (contig [0492] and contig [5143]) were also up-regulated. In the *Caenorhabditis elegans*-Cry5B interaction, 106 *hpo* (hypersensitive to pore-forming toxin) genes were important for cellular protection against an attack because knock-downs showed hypersensitive to Cry5B phenotype [18]. For example, catalase (ctl-2) gene functions as an antioxidant enzyme that protects cells from reactive oxygen species, and ctl-2 expression is negatively regulated by DAF-2 mediated insulin signaling. The DAF-2 mediated insulin signaling pathway has been identified as one of the cellular defense mechanisms in *C. elegans* [38]. These transcripts (contig [0492] and contig [5143]) may be involved in immune defense by accelerating infected cell death, and triggering the intracellular daf-2 insulin pathways in response to the ingestion of Cry1Ab protoxin. 

In this study, we also observed a 2.7-fold decreased expression of a chitinase transcript (contig [0188]) and 2.2-fold increased expressions of both chitin synthase 2 (ECB-V-28_H03) and glucosamine-fructose-6-phosphate aminotransferase (GFAT) 2 (ECB-C-05_D05) transcripts. A gut specific chitinase gene has been reported to play an important role in regulating the chitin content of peritrophic matrix in the midgut of *O. nubilalis* larvae [39]. On the other hand, chitin synthase 2 has been known to be specifically responsible for biosynthesis of chitin associated with peritrophic matrix in the midgut [40,41,42,43], whereas GFAT catalyzes the formation of glucosamine 6-phosphate and is an important enzyme in chitin biosynthetic pathway [41]. Thus, the down-regulation of the gut chitinase gene and the up-regulation of chitin synthase 2 and GFAT genes may act as a defense mechanism to maintain the gut system integrity in response to the Cry1Ab ingestion in the larvae.

In summary, our microarray analysis of the transcript expression in the gut of *O. nubilalis* larvae in response to the ingestion of Cry1Ab protoxin has shown many interesting phenomena that likely reflect physiological changes. However, this study is a preliminary survey of Cry1Ab protoxin induced transcriptional responses in *O. nubilalis*, and many hypotheses generated in this study require further validations and analyses by using additional approaches in order to better understand the molecular basis of the Bt protoxin and gut interactions in this important insect pest. 

## 3. Materials and Methods

### 3.1. European Corn Borer

The Bt-susceptible strain (Lee) of *O. nubilalis* was obtained from French Agricultural Research, Inc. (Lamberton, MN, USA). Larvae were reared at 26 °C using artificial diet (Bio-Serv, Frenchtown, NJ, USA), and adults were reared in a metal cage under long-day conditions (L:D = 16:8) and 70% humidity, and routinely fed 2% sucrose water to provide supplementary reproductive nutrition. The eggs were collected on wax paper every day and kept in insect rearing cups with high humidity (≥80%) until hatching. Newly hatched larvae were immediately transferred to artificial diet and reared to the third instar for testing. The larval developmental stage was monitored by moving larvae to a new rearing dish after each molt.

### 3.2. Determination of Median Lethal Concentration of Cry1Ab Protoxin in 7-Day Bioassay

Cry1Ab protoxin was prepared from *Escherichia coli* (strain ECE54) which harbor cry1Ab gene based on the previously described method [44], and stored at −80 °C as a suspension until use. The bacterial strain was provided by the *Bacillus* Genetic Stock Center, Ohio State University (Columbus, OH, USA). The median lethal concentration (LC_50_) of Cry1Ab protoxin was determined in a 7-d bioassay at room temperature. In this assay, third-instar larvae were starved for 24 h, and larvae were fed artificial diet containing no Cry1Ab protoxin (0 µg/mL) as a control and each of five concentrations of Cry1Ab protoxin (0.04, 0.20, 1.0, 5.0, and 25 µg/mL) as a treatment. Each control or treatment was repeated three times and 16 starved larvae were used in each control or treatment. Fresh artificial diet containing Cry1Ab protoxin was replaced every other day, and surviving individuals were recorded every day. The bioassay data were analyzed by probit analysis using PROC GLM procedure. After 7 d, the LC_50_ for the *O. nubilalis* larvae was determined to be 0.25 µg/mL (95% CI = 0.14–0.33 µg/mL). Mortality was not observed in the control larvae fed artificial diet only.

### 3.3. Cry1Ab Protoxin Bioassay

For this experiment 30 third-instar larvae were first starved for 24 h to ensure that they would feed immediately when placed on experimental diets. The larvae were divided into two groups (control and treated) and each group consisted of three replicates (5 individuals in each replicate). The larvae in the treated group were fed artificial diet containing the concentration of 0.25 μg of Cry1Ab protoxin per ml of diet. Cry1Ab protoxin was first dissolved in 50 mM sodium carbonate buffer, pH 10.0 [14]. The larvae in the control group were fed protoxin-free diet as described by van Munster *et al.* [21]. This concentration of Cry1Ab protoxin was the LC_50_ observed in a preliminary 7-day bioassay for third instar larvae as described above. Aliquots of 100-µL liquid diet with or without Cry1Ab protoxin were loaded into a 96-well microplate. The diet was allowed to solidify for 30 min at room temperature. The starved larvae were individually transferred into the wells and allowed to feed on the respective diets for 6 h. The larva was collected from each well and dissected to obtain the whole gut. A total of five whole guts were pooled as a sample for total RNA extraction. The 6-h feeding time was based on the observation that larvae had stopped feeding, but there were as yet no visible effect and larval mortality. Therefore, the concentration of 0.25 μg of Cry1Ab protoxin per ml of diet was close to the no observed effect concentration (NOEC) for the 6-h feeding period. Total RNA was prepared independently for each replicate (a group of five larvae) using TRIzol reagent (Invitrogen Inc., Frederick, MD, USA). The quantity and quality of the total RNA were determined by NanoDrop 2000 spectrophotometer (Thermo Fisher Scientific Inc., Waltham, MA, USA) and Agilent 2100 bioanalyzer (Agilent Technologies Inc., Santa Clara, CA, USA). 

### 3.4. Microarray Analysis

We previously sequenced 15,000 expressed sequence tags (ESTs) from the larval gut of *O. nubilalis* [17]. A total of 12,519 high quality ESTs with an average length of 656 bp were deposited in the EST database (dbEST) with GenBank accession numbers from GH987145 to GH999663 at the NCBI. A high-resolution 8 × 15 K multi-pack expression microarray for single-color detection was designed using Agilent’s probe design algorithms (Agilent). In average, five oligonucleotide probes from each of 2895 unique ESTs from the *O. nubilalis* larval gut were computationally designed, and potential cross-hybridization probes were discarded; however, some transcripts only have less than five probes after a cross-hybridization examination. In total, 12,972 usable probes were obtained from *O. nubilalis* ESTs, which represent 2895 unique gut transcripts [17]. Agilent also provided the background control and the standard control probes. Agilent’s sure-print inkjet technology was employed to directly synthetize all oligo probes (60 mers) on specially prepared glass slides. Each glass slide contained eight identical microarray chips. The datasets of the gene expression profiles have been deposited in the NCBI Gene Expression Omnibus (GEO) repository with the accession number of GSE55685 (http://www.ncbi.nlm.nih.gov/geo/query/acc.cgi?acc=GSE55685).

**Figure 3:**
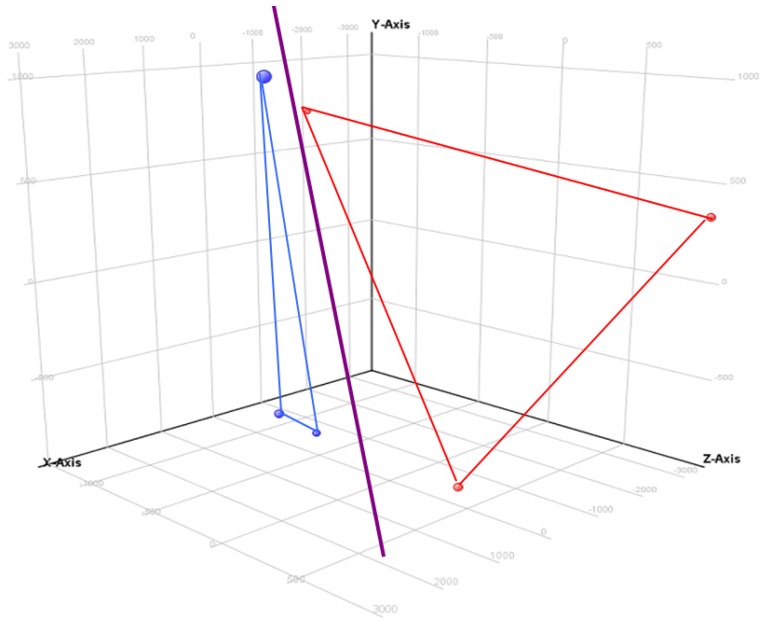
Microarray data quality control test using principal components analysis (PCA) plot. The blue spots indicated three samples from the larvae fed the diet with Cry1Ab protoxin, whereas the red spots represented three samples from the larvae fed the diet with on protoxin.

Cyanine-3 labeled cRNAs were synthesized from Agilent single-color microarray-based gene expression kit. Dye-incorporation ratio was determined using NanoDrop 2000 spectrophotometry. The cRNA samples with the ratio cyanine-3 labeled cRNA ≥10 pmol/µg were used for hybridization. Cyanine-3 labeled cRNAs (600 ng) of each sample was hybridized to the microarray chip (six samples, including 3 from the control diet and 3 from the protoxin diet, and were hybridized on six individual microarray chips) and incubated at 65 °C for 17 h. Slides were scanned using an Axon GenePix 4000B (Molecular Devices Inc., Sunnyvale, CA, USA) microarray scanner at a 532 nm wavelength. The signal intensity of each hybridized spot was qualified and quantified with Agilent Feature Extraction Ver. 9.5 software (Agilent). At beginning, six raw data files with 12,972 customer designed probes and control probes extracted by Agilent Feature Extraction software were imported to GeneSpring GX11 and were applied “75% percentile shift” normalization algorithm. Six samples were grouped into two groups (three controls and three treatments). The quality control was assessed by examining principal components analysis (PCA) plots and correlation analysis of sample replicates. The correlation coefficients within Cry1Ab treatment or control group were larger than 97% indicating the high quality control (Figure 3). Analysis of variance (one-way ANOVA, p < 0.05) was performed to test the variation between two groups. The Benjamini-Hochberg multiple testing correction (q < 0.05) was also employed after ANOVA to identify the transcripts that were differentially expressed when the cutoff of the fold change was set at ≥2.0. The expression differences with p < 0.05 and with expression ratios ≥2.0 were considered significantly different [22,45]. A total of 758 probes were identified to be differentially expressed at the significant level as specified. These probes represented 174 unique transcripts found in the larval gut. Finally, gene ontologies of 174 transcripts were analyzed by using Blast2go (http://www.blast2go.org, BioBam Bioinformatics S.L. Valencia, Spain) at level 2. 

### 3.5. Validation of Expression Changes by RT-qPCR

Before the expression changes were validated by RT-qPCR, the efficiency of each primer pair was evaluated first and only one unique product (one peak in the efficiency graph for each primer pair) was obtained. The efficiencies of the primer pairs used in our RT-qPCR analysis ranged from 95% to 110% by using whole gut cDNA from *O. nubilalis* as a template. The total RNA used for microarray analysis was also used for RT-qPCR analysis. One microgram of total RNA was reverse-transcribed in a 20-µL reaction mixture using Fermentas ReverAid^TM^ First Strand cDNA synthesis kit (Fermentas Inc., Glen Burnie, MD, USA). RT-qPCR was performed in a Bio-Rad iCycler (Bio-Rad Laboratories, Hercules, CA, USA) by using Fermentas SYBR green qPCR kit (Fermentas). Quantitative PCR was performed with 2-step amplification protocol with 40 cycles of 95 °C for 30 s, 56 °C for 30 s using a Bio-Rad IQ thermocycler (Bio-Rad Laboratories Inc. Hercules, CA, USA). The specific primers for 13 candidate genes as inferred from other studies [14,24,25] and the endogenous reference gene (*ribosome protein L18*, *OnRpl18*) were designed using Beacon 7 Designer^TM^ (Table 2). The abundance of each transcript was normalized to *OnRpl18*, using the ∆Ct equation (Ct (target)—Ct (reference)). The relative abundance of each transcript in the treatment (Cry1Ab protoxin) compared with the controls was calculated using the ∆∆Ct relative expression (2^−(∆Ct treatment−∆Ct control)^) method [46], and the significantly different expression of each transcript was evaluated by student *t*-test (*p* ≤ 0.05). 

**Table 2:** Sequences of primers used for reverse transcription quantitative PCR (RT-qPCR) analysis.

Gene name	Primer sequences	Product size (bp)	EST ID
Trypsin-like serine protease	GGACAGTTCTCTGAGCAGTTAC	109	contig [4786]
	ACAGCATGTTGTCAGTGATGG		
Trypsin-like serine protease	ATTCTCAACAACAGGGCTATTTTG	148	contig [3704]
	TGTAGTCAGGGTGGTTAATGATTC		
Trypsin-like serine protease	GCATCATACCCGTCACATCTAC	148	contig [0770]
	GTGAAGTTGCCGTACTGAGTC		
Trypsin precursor	GCCAGCATTACACCTTCCG	128	contig [0243]
	TCGCAGTTCTCGTAGTAAGAC		
Silk gland derived trypsin serine protease	CACAAAGTCCTGGAGGAAGATTC	125	ECB-C-18-B11
	GTTCACGCCTGTCTGTTGC		
Chymotrypsin-like serine protease	GGTGCTTGTTAGTATGTT	116	contig [0389]
	AAACTTCTTTAATTGCTCAG		
Chymotrypsin-like serine protease	ATAGAGCACCCGAATTACAACG	123	contig [1207]
	GTAGGTTTGCGAGCCAGTG		
Chymotrypsin-2	CCCCTTCGTCCACGCTAG	123	contig [5740]
	GTCACACCAACCAAGAGTCTC		
Aminopeptidase N	TTCCAAACACATTTTCTTG	118	contig [4776]
	AAGCGTATTGTCCTCTAT		
Aminopeptidase N	CAGTAGCGATAACATCAC	183	contig [4879]
	CCAGTCAAGTCTTCTCTA		
Aminopeptidase N	GTCAACGAAATTGTCATC	109	ECB-V-05-D12
	AGTCATATTCTGGCTGTA		
Cadherin-like protein	CTATGTGTTCTCAATCCAA	75	J-ECB-25-B09
	TCGTCGATGTTGACTATC		
Alkaline phosphatase	CGGATTATCTGCTGGGTTTATTTG	79	contig [5858]
	AGTGTGGGCTCGGTAACG		

## Supplementary Files

Supplementary File 1 Supplementary Information (PDF, 96 KB)
