# Supplementary material for: Changes in Gene Expression in the Larval Gut of Ostrinia nubilalis in Response to Bacillus thuringiensis Cry1Ab Protoxin Ingestion
Source: Toxins (Basel). 2014 Apr 3;6(4):1274–94. doi: 10.3390/toxins6041274 (PMC4014733; doi:10.3390/toxins6041274)
Supplement: Supplementary File 1 — Supplementary Information (PDF, 96 KB) [file toxins-06-01274-s001.pdf]

Supplementary Information

Supplementary Table S1. Summary of 56 significantly differentially expressed (fold change ≥2.0 and $p < 0.05$) transcripts without BLAST results from *O. nubilalis* larvae in response to the ingestion of Cry1Ab protoxin.

EST ID	NCBI EST database ID	FC (fold change)
Contig[1263]	GH996374.1	4.74
Contig[1469]	GH993948.1	2.37
Contig[1573]	GH997739.1	2.07
Contig[2558]	EL929309.1	3.76
Contig[3166]	GH990512.1	−2.50
Contig[3324]	GH990723.1	2.04
Contig[3688]	GH998654.1	2.52
Contig[4380]	GH988114.1	2.21
Contig[5262]	EL929327.1	5.52
Contig[5443]	GH995614.1	2.10
Contig[5770]	GH989043.1	2.28
Contig[5860]	GH996959.1	2.03
Contig[5929]	GH990692.1	2.60
Contig[6000]	GH998860.1	2.14
Contig[6008]	GH988209.1	3.19
ECB-05_M13R_D01	GH997516.1	2.55
ECB-06_M13R_H09	GH997649.1	2.17
ECB-10_M13R_E05	GH997908.1	−3.00
ECB-12_M13R_A06	GH998037.1	−3.51
ECB-14_M13R_E05	GH998249.1	−3.62
ECB-14_M13R_H12	GH998287.1	2.13
ECB-15_M13R_H03	GH998369.1	3.06
ECB-18_M13R_B07	GH998575.1	3.99
ECB-20_M13R_C07	GH998765.1	2.75
ECB-25_M13R_E04	GH999198.1	−2.46
ECB3_M13F_G02	GH996861.1	−3.05
ECB4_M13F_C06	GH996901.1	−2.14
ECB-C-07_M13R_B08	GH993088.1	2.60
ECB-C-11_M13R_A06	GH993413.1	−2.86
ECB-C-11_M13R_C06	GH993433.1	2.46
ECB-C-18_M13R_H11	GH994018.1	2.52
ECB-V-10_M13R_B03	GH994962.1	−2.30
ECB-V-14_M13R_F02	GH995309.1	2.78
ECB-V-16_M13R_B02	GH995433.1	2.36
ECB-V-21_M13R_E05	GH995863.1	−3.26
ECB-V-22_M13R_F04	GH995951.1	2.04
gi_133906575	EL929413.1	9.88
gi_133906598	EL929436.1	13.3
gi_133906621	EL929458.1	3.67
gi_133907083	EL929911.1	3.10

Supplementary Table S1. *Cont.*

EST ID	NCBI EST database ID	FC (fold change)
J-ECB-02_M13R_D07	GH989166.1	2.20
J-ECB-07_M13R_D07	GH991658.1	2.23
J-ECB-12_M13R_B11	GH989392.1	2.19
J-ECB-21_M13R_D12	GH988931.1	24.1
J-ECB-25_M13R_H03	GH991098.1	4.21
J-ECB-29_M13R_A11	GH992338.1	2.35
J-ECB-40_M13R_B04	GH987796.1	2.34
J-ECB-45_M13R_B11	GH989931.1	2.21
J-ECB-45_M13R_C07	GH989976.1	2.26
J-ECB-49_M13R_D08	GH991956.1	2.02
J-ECB-51_M13R_E01	GH987763.1	3.37
J-ECB-51_M13R_H01	GH987821.1	2.98
J-ECB-53_M13R_C06	GH988091.1	−2.22
J-ECB-56_M13R_F08	GH989599.1	3.41
J-ECB-57_M13R_E02	GH990005.1	−3.27
J-ECB-61_M13R_C07	GH987258.1	2.39

© 2014 by the authors; licensee MDPI, Basel, Switzerland. This article is an open access article distributed under the terms and conditions of the Creative Commons Attribution license (http://creativecommons.org/licenses/by/3.0/).
